# Development of an *in vitro* system to study oral biofilms in real time through impedance technology: validation and potential applications

**DOI:** 10.1080/20002297.2019.1609838

**Published:** 2019-05-06

**Authors:** Alex Mira, Elena Buetas, Bob Rosier, Danuta Mazurel, Álvaro Villanueva-Castellote, Carmen Llena, Maria D. Ferrer

**Affiliations:** a FISABIO Foundation, Centre for Advanced Research in Public Health, Valencia, Spain; b Departamento de Estomatologia, University of Valencia, Valencia, Spain

**Keywords:** Oral biofilms, multiple-species biofilm, *in vitro* model, real-time, biofilm dynamics, impedance, dental plaque, tongue, antibiotic, Streptococcus mutans

## Abstract

**Background and objectives**: We have developed a standardized, easy-to-use in *vitro* model to study single- and multiple-species oral biofilms in real time through impedance technology, which elucidates the kinetics of biofilm formation in 96-well plates, without the requirement for any further manipulation.

**Design and Results:** Using this system, biofilms of *Streptococcus mutans* appear to be sugar-dependent and highly resistant to amoxicilin, an antibiotic to which this oral pathogen is highly sensitive in a planktonic state. Saliva, tongue and dental plaque samples were also used as inocula to form multiple-species biofilms. DNA isolation and Illumina sequencing of the biofilms showed that the multi-species biofilms were formed by tens or hundreds of species, had a similar composition to the original inoculum, and included fastidious microorganisms which are important for oral health and disease. As an example of the potential applications of the model, we show that oral biofilms can be inhibited by amoxicilin, but in some cases they are induced by the antibiotic, suggesting the existence of responders and non-responders to a given antibiotic.

**Conclusions:** We therefore propose the system as a valid *in vitro* model to study oral biofilm dynamics, including their susceptibility to antibiotics, antiseptics or anti-adhesive compounds.

Microorganisms colonizing the oral cavity are attached to surfaces (soft or hard) and live in complex microbial communities termed biofilms. Its inhabitants, which may be made up of a network of hundreds of different species, are surrounded by extracellular polymeric substances conferring protection against external factors such as antimicrobial agents, facilitating their survival [1]. Within oral biofilms, dental plaque is probably the best studied. In this case, the biofilm is built upon a hard and inert surface, the tooth, which is constantly being irrigated by saliva. In fact, it has been established that for the first phase of dental plaque development, a layer of saliva binds to enamel to form the so-called acquired film/pellicle whose proteins and host receptors influence bacterial adhesion [2]. Furthermore, the oral microbiota also colonizes soft surfaces such as the tongue, where resident communities vary according to anatomical locations [3]. Nowadays, it is known that in the oral cavity both bacterial diversity and abundance are diverse in each biofilm location, and even within each location there are microniches that condition and restrict microbial adaptation [4]. Likewise, oral diseases of polymicrobial etiology such as caries (tooth decay), periodontitis (gum disease) and halitosis (bad breath), are actually the result of a dysbiosis in which a healthy biofilm turns into a pathogenic one [5–7]. In this regard, bacterial interactions (co-adhesion) required to form each type of biofilm according to its location, are probably different, as well as its formation dynamics [8].

Different *in vitro* model systems have been developed to study complex biofilms. Oral biofilm models can roughly be divided into those that use natural samples such as saliva or dental plaque [9,10], or approaches in which defined bacterial populations are employed [11]. The biofilm models that most closely resemble *in vivo* microbial communities are certainly those that apply real samples with whole ecosystem diversity, but these microcosms are also more complex to interpret and standardize due to biological heterogeneity. From the point of view of the equipment or device used to study biofilms, two approaches have been developed. The first ones are open systems, with continuous supply of nutrients or flow conditions [12,13]. The main advantage of this equipment is that it simulates *in vivo* conditions more faithfully, but they are usually laborious and expensive tools, and above all do not allow testing several factors simultaneously. A second kind of systems for biofilm studies are the so-called batch devices. These gadgets are closed culture systems, with different surfaces for oral biofilm growth such as multi-well plates [14], pegs [15] or hydroxyapatite discs [16], which are widely employed in oral biofilms studies for being easy to use, high reproducibility and high yield, as well as allowing different factors to be screened at one time in the same experiment.

Every device has advantages and disadvantages but a generalized weakness of most available systems is that they do not allow the study of biofilms in real time; instead, results are observed only at a particular timepoint, with the consequent loss of information. To solve this shortcoming, microscopy analysis can be adapted to visualize biofilm development [17,18]. For example, combining different fluorescence *in situ* hybridization probes (FISH) with confocal laser scanning microscopy (CLSM) adapted to a flow cell biofilm model can allow monitoring biofilm architecture continuously and discern each probe-labeled species’ spatial location [19]. In summary, there are sophisticated systems available for studying biofilms in real time, but they normally involve a high processing time to observe the results, along with delicate sample handling. In addition, they are usually expensive devices for performing multiple replicates or for high-throughput application. The aim of this work is to present a new model to study oral biofilms, including those formed by multiple-species, in real time. The methodology we have developed is based on single-frequency impedance spectroscopy, which allows quantifying single-species biofilm growth while also providing information about the kinetics of its formation, without the requirement for any further manipulation [20,21]. In this work, the system is applied to individual oral pathogenic species as well to complex biofilm formation from various kinds of inocula from oral samples, such as saliva, tongue and plaque from the tooth surface or the gingival sulcus. We used DNA extracted from both the biofilm grown *in vitro* and the original sample for PCR amplification and Illumina sequencing, in order to test whether the biofilm formed is a multi-species biofilm of composition and abundance similar to the original inoculum. Finally, we illustrate the potential applications of this finding by testing the amoxicillin antibiotic effect on biofilm formation dynamics of oral samples from different donors.

## Materials and methods

### Real-time single-species biofilm analysis

Real-time biofilm assays were performed with an xCELLigence RTCA SP [SP = single plate] instrument according to the instruction of the supplier (ACEA Biosciences) [22]. *Streptococcus mutans* ATCC 25,175 was grown overnight in BHI and diluted in BHI or BHI supplemented with 0.1% sucrose (BHI-sucrose), to OD650 = 0.03. For monitoring biofilm formation and RTCA sensitivity assays, 100 µl of BHI (Brain Heart Infusion medium) or BHI-sucrose was added to each well of non-reusable 96X microtiter E-plates (ACEA Biosciences) and impedance background measurement was performed following the standard protocol (described in ACEA’s Application Note No. 17, Studying Bacterial Biofilms Using Cellular Impedance [23]). A 100 µl sample of the cell suspension was then added to the 96 E-plate wells. Each sample was run in triplicate. E-plates were positioned in the xCELLigence Real-Time Cell Analyzer SP, incubated at 37ºC and monitored on the RTCA system at 10-min time intervals for 24 h. Cell-sensor impedance was expressed as an arbitrary unit called Cell Index (CI) according to the manufacturer’s instructions, which represents a measure of total biofilm mass [20]. The CI at each timepoint taken at 10 kHz frequency is defined as (Z_n_-Z_b_)/F, where Z_n_ is the cell-electrode impedance of the well when it contains cells. Z_b_ is the background impedance with growth media alone, and F is related to 10Ω. Standard deviations of duplicates or triplicates of wells were analyzed with the RTCA Software.

### Donor selection and sampling procedure

All the volunteers selected for sampling had not been treated with antibiotics in the 3 months prior to the study nor did they report antecedents of routine use of oral antiseptics. The written informed consent signed by volunteers as well as the sampling procedure were approved by the Ethics Committee from the DGSP-CSISP (Valencian Health Authority), with reference 270,516.

For experiments of biofilm formation with saliva, tongue and supragingival plaque, four donors were selected (D2, D3, D5 and D6). They were two men and two women, aged 20–40 years, non-smokers, with 28 teeth excluding third molars, and in good dental and periodontal health (no active caries lesions, no periodontal disease). All samples were taken in the morning, 24 h after toothbrushing, in the following order: donors provided a 2-mL non-stimulated, drooling saliva sample in a 50 ml sterile Falcon tube; samples from the tongue dorsum were then collected with an autoclaved spoon excavator across the entire surface, with several repetitive strokes to ensure a representative sample; supragingival dental plaque samples were taken from vestibular (buccal) and lingual (palatine) surfaces from the first incisor, canine, first premolar and first molar from each quadrant. Teeth were not dried before sampling and each sample was taken with a different sterile spoon excavator.

For saliva samples taken before and after a food intake, two men aged 30–40 years with the above-mentioned oral health conditions were selected. In this assay, non-stimulated drooling saliva samples were provided just before and 15 min after a food intake.

For subgingival plaque sampling, four different volunteers (SP1, SP2, SP3 and SP4) were selected based on periodontal health. SP1 and SP2 donors showed good dental and periodontal health. SP3 had gingivitis and periodontal pockets of 3–4 mm, and SP4 had advanced periodontal disease with periodontal pockets >6 mm. Subgingival samples were taken from the vestibular and lingual surfaces from the first incisor, canine, first premolar and first molar from each quadrant., by means of two sterile absorbent paper points (size 25) per sample, introduced into the gingival sulcus for one min, avoiding contact with the supragingival dental plaque.

Tongue, supragingival and subgingival plaque samples were inserted into a tube with BHI-sucrose and after homogenizing by vortex to break the aggregates, were immediately processed to study its biofilm formation.

### Real-time multi-species biofilm analysis

Following impedance background measurement with 100 ul of BHI-sucrose, 100 ul of each sample type were added to each well. For saliva biofilms, 100 ul of non-stimulated saliva were inoculated directly per well after the background measurement. In the case of tongue, supragingival and subgingival plaque samples, 100 ul of each sample suspension previously homogenized in BHI-sucrose (see above) were added per well of non-reusable 96X microtiter E-plates (ACEA Biosciences). Each sample was run in triplicate. To generate anaerobic growth conditions, 50 ul of mineral oil (Sigma M8410) was overlaid on top of the growth media in each well prior to positioning the plate in the xCELLigence Real-Time Cell Analyzer SP to start monitoring biofilm formation. The rest of the procedure for monitoring multi-species biofilm formation was similar to the one for single-species biofilms (described above).

### Quantification of antibiotic effect on the oral biofilm

For evaluating the effectiveness of amoxicillin to inhibit biofilm formation, 100 µl of each antibiotic dilution (32 µg ml^−1^, 16 µg ml^−1^, 4 µg ml^−1^, 1 µg ml^−1^, 0.25 µg ml^−1^ and 0.13 µg ml^−1^) in BHI-sucrose were added to measure background impedance. Then, 100 µl of saliva or the *S. mutans* suspension (OD650 = 0.03) were added, respectively. Two replicates of each antibiotic concentration and negative controls without antibiotic were also included. The lowest antibiotic concentration that inhibited biofilm formation with a CI value <0.05 was considered the Biofilm Minimum Inhibitory Concentration (MBIC).

For evaluating the effectiveness of amoxicillin on already-formed *S. mutans* biofilm, background impedance measurement was performed with a 100 µl of an *S. mutans* overnight culture diluted in BHI-sucrose to OD650 = 0.015. After that, an additional 80 µl of this cell suspension was added and monitored on the RTCA system for 18 h. Plates were then removed from the incubator and 20 µl of antibiotic dilutions were added (reaching a final concentration of 32 µg ml^−1^, 16 µg ml^−1^, 4 µg ml^−1^, 1 µg ml^−1^, 0.25 µg ml^−1^ and 0.13 µg ml^−1^). Cells were then monitored for another 24 h. Each antibiotic concentration was tested in duplicate, in addition to negative controls which lacked antibiotic. For data analysis, CI values were normalized by subtracting the negative control impedance, and the average of two replicates was calculated for plotting the CI graphs.

### Biofilm removal and DNA extraction

At the time in which the highest CI value was achieved for each different sample type, the biofilm adhering to the bottom surface of the well was removed as follows: After taking the plate out from the incubator, the supernatant was extracted from the well. Next, two gentle washes with 150 ul of phosphate buffer were performed to remove non-adhered cells. Subsequently, two further exhaustive washes focusing on the bottom surface were carried out until complete dispersion of all adhered material was achieved. Disaggregated biofilm was recovered and stored at −20ºC until processed.

DNA was extracted with the MagnaPure LC JE379 instrument and the MagnaPure LC DNA Isolation Kit (Roche) following the manufacturer´s instructions with some modification to include a strong enzymatic lysis. Specifically, the additional enzymatic lysis step included lysozyme (20 mg/ml, 37°C, 60 min), lysostaphin (2,000 units/mg protein, 37°C, 60 min) and mutanolysin (4,000 units/mg protein, 37°C, 60 min), following reference [24]. The obtained DNAs were eluted in 100 µl of nucleic acid-free water and quantified by fluorometry with the Qubit Kit (Invitrogen).

### 16S rRNA *gene amplification and sequencing*

An Illumina amplicon library was performed following the *16S rRNA* gene Metagenomic Sequencing Library Preparation Illumina protocol (Part #15044223 Rev. A). The gene-specific primer sequences used in this protocol target the *16S rRNA* gene V3 and V4 regions, resulting in a single amplicon of approximately 460 bp [24]. Overhang adapter sequences were used together with the primer pair sequences for compatibility with Illumina index and sequencing adapters. After *16S rRNA* gene amplification, the DNA was sequenced on a MiSeq Sequencer according to manufacturer’s instructions (Illumina) using the 2 × 300 bp paired-end protocol.

Only overlapping paired-end reads were used for analysis. A sequence quality assessment was carried out using the PRINSEQ program [25]. Sequences of <350 nucleotides in length were not considered; sequence end-trimming was performed by cutting out nucleotides with a mean quality of <30 in 20-bp windows. Chimeric *16S rRNA* gene sequences were filtered out using the USEARCH program [26]. Obtained sequences were taxonomically classified by the RDP-classifier [27] where reads were assigned at the genus level.

### Statistical analysis

Constrained (canonical) correspondence analysis (CCA) was used to emphasize variation between samples, under the hypothesis that sample source can explain part of the total variability in the data. The analysis was performed by the R software, using the ade4 package [28] with the CCA function, based on Chi-squared distances. Adonis statistic for permutational multivariate analysis was used to measure differences in variance between groups using the R library ‘vegan‘ [29].

## Results

### Mono-species biofilm formation is sugar-dependent

**Figure 1.**
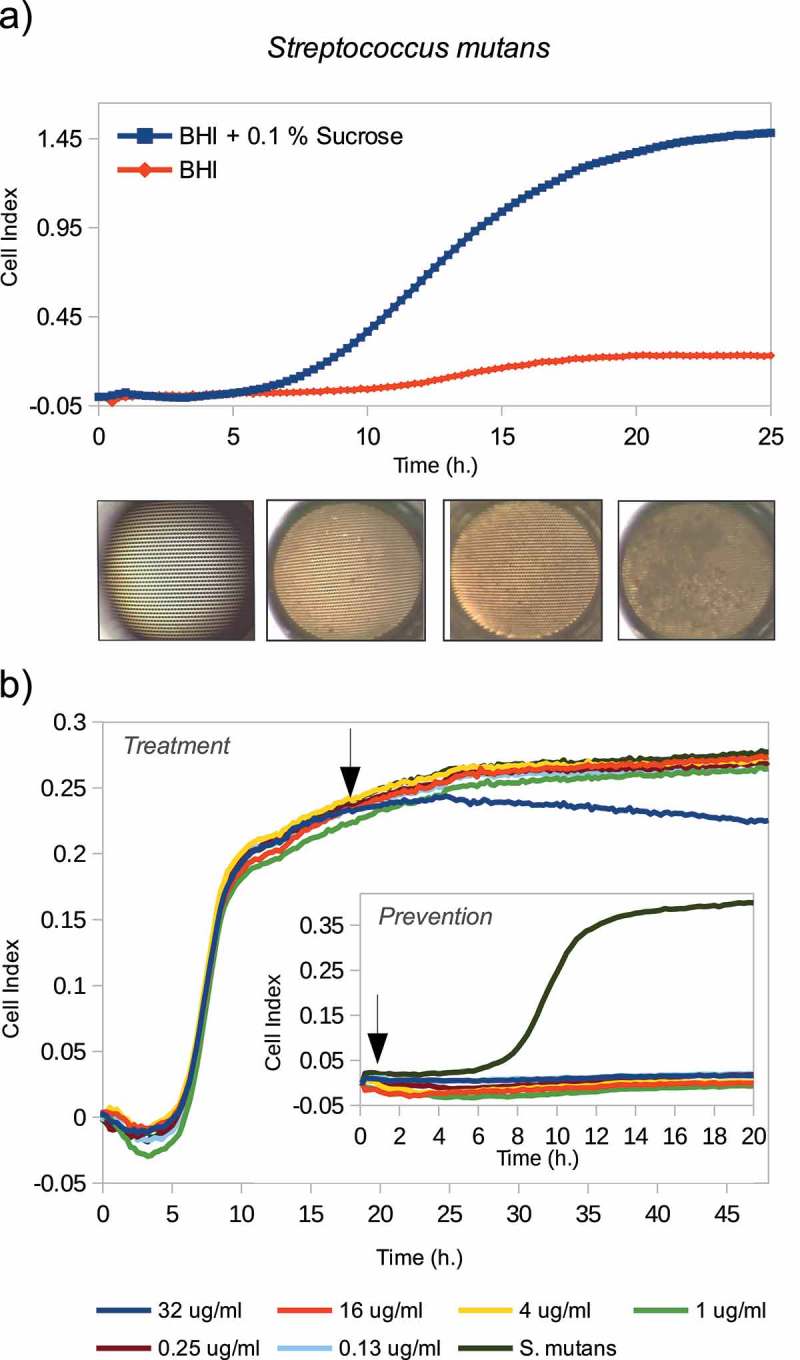
(a)*S. mutans* biofilm formation monitored in real time using cell impedance measurements, grown in both BHI culture medium (in red) and BHI plus 0.1% sucrose (in blue) for 25 h. Below the graph, photographs of the bottom of the well taken at 0, 6, 12 and 24 h of *S. mutans* biofilm formation growing in BHI medium plus 0.1% sucrose. (**b**) Amoxicillin effect on the *S. mutans* biofilm formation measured with the RTCA xCELLigence system. Arrows indicate when different antibiotic concentrations were added: from the beginning along with the cells (internal graph), and once the biofilm was already formed (external graph).

*S. mutans*, a dental plaque community member, plays a key role in modulating the transition from a non-cariogenic biofilm to one with a high cariogenic potential [30]. Knowing that the synthesis of structural glucans is increased in the presence of sucrose, which provides enhanced bacterial binding to surfaces [31,32], the effect of sucrose addition to the culture medium (BHI + 0.1% sucrose) on *S. mutans* mono-species biofilm formation was studied. Figure 1(a) shows the impedance measurements generated every 10 min, represented as Cell Index values (CI), during the growth of *S. mutans* adhered to the bottom of the well, in medium with and without sucrose. Photographs of the bottom of the well taken at different times of *S. mutans* biofilm growth (6, 12 and 24 h) in the presence of sucrose are shown below the graph (Figure 1(a)). A pattern of stripes corresponding to the gold electrodes can be observed at the bottom of each well, being more difficult to appreciate after 24 h of biofilm growth. During the first 6 h of biofilm formation with sucrose, an increase in CI values was not observed, which could correspond to the first adhesion stage. Then, an exponential growth phase (cell proliferation and extracellular matrix generation) was identified, reaching the plateau or stationary phase at approximately 20 h of monitoring, which was maintained until at least 25 h.

Oral diseases are currently considered to have a polymicrobial etiology, and the most commonly used antibiotic therapy of choice is amoxicillin-clavulanic acid [33]. Classic antibiotic susceptibility assays routinely used in clinical practice are those based on dilution or diffusion methods designed for bacteria in planktonic life or agar plates. However, the standard MICs are often insufficient to inhibit biofilms, especially if they are mature, and in some instances, it may even induce their overgrowth [20]. In this regard, we have determined the *in vitro* amoxicillin effect at different concentrations (32 μg/ml, 16 μg/ml, 4 μg/ml, 0.25 ug/ml and 0.13 ug/ml) on *S. mutans* biofilm formation, both when the antibiotic is added at the beginning of the experiment together with the inoculum (i.e. simulating an antibiotic preventive effect), and adding it once the biofilm is already formed. Arrows in the graphs of Figure 1(b) show the time at which the antibiotic is added, both on the mature biofilm (external graphic) and before it is formed (internal graph), respectively. As shown in the internal graph of Figure 1(b), when the antibiotic is added from the beginning of the experiment, all amoxicillin concentrations tested inhibit *S. mutans* biofilm formation. This could be due to inhibition of biofilm growth or more probably to a lower amount of viable bacteria. By contrast, no concentration was able to inhibit or disaggregate the fully formed biofilm, although the highest concentration (32 μg/ml) appears to stop further growth.

### Quantifying multi-species biofilm formation

**Figure 2.**
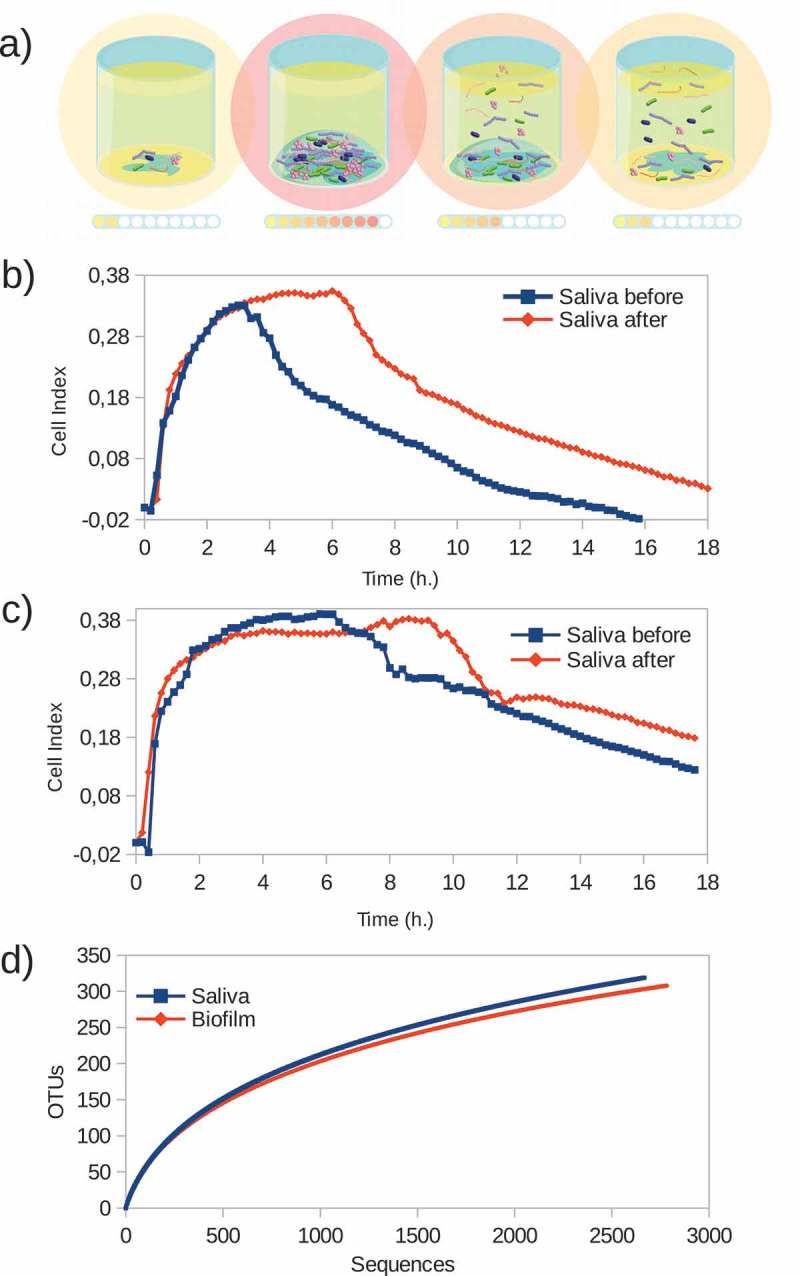
Monitoring saliva samples biofilm formation in real time. (**a**) Schematic representation of successive biofilm development stages on the impedance system wells. Intensity of the impedance measurements in each step is represented by colored spots. (**b**) and (**c**) Biofilm formation (represented as Cell Index values, or CI) graphs from two unstimulated saliva samples taken before (blue line) and after (red line) a food intake of two volunteers. (**d**) Species richness estimation by rarefaction curves of both adhered biofilm and corresponding saliva sample.

In order to assess whether the RTCA system allows the formation of a complex biofilm representative of natural ecosystems, a pilot experiment with saliva samples was performed. Figure 2(b,c) show biofilm formation CI graphs obtained from two unstimulated saliva samples taken before and after a food intake of two volunteers. During the first 2 h a sharp biofilm growth can be detected, suggesting that cell attachment and initial development of saliva biofilm architecture are very fast. Then, CI reached a plateau, whose length is donor-specific (Figure 2(b,c)). This phase was maintained for two more h in post-meal saliva samples of both volunteers, perhaps related to an increased amount of nutrients or higher microbial activity. Finally, the CI values begun to decrease slowly, probably due to changes in nutrient levels that induce cellular detachment. The different phases observed in CI patterns through the RTCA system therefore resemble the different biofilm development stages: adherence, maturation and disassembly, which may allow quantification of their dynamics (Figure 2(a)).

To investigate the diversity of the biofilm formed from the saliva samples, bacterial composition of the biofilms was determined by *16S rDNA* sequencing and compared with the composition of the inoculating sample. Rarefaction curves shown in Figure 2(d) represent the estimated species richness of adhered biofilms and corresponding saliva samples, demonstrating that the biofilm formed on the surface of the RTCA wells is made up of a species number similar to the inocula, and therefore does not derive from a limited number of bacteria specially adapted to grow under these conditions.

### Multi-species biofilms from different sample types

**Figure 3.**
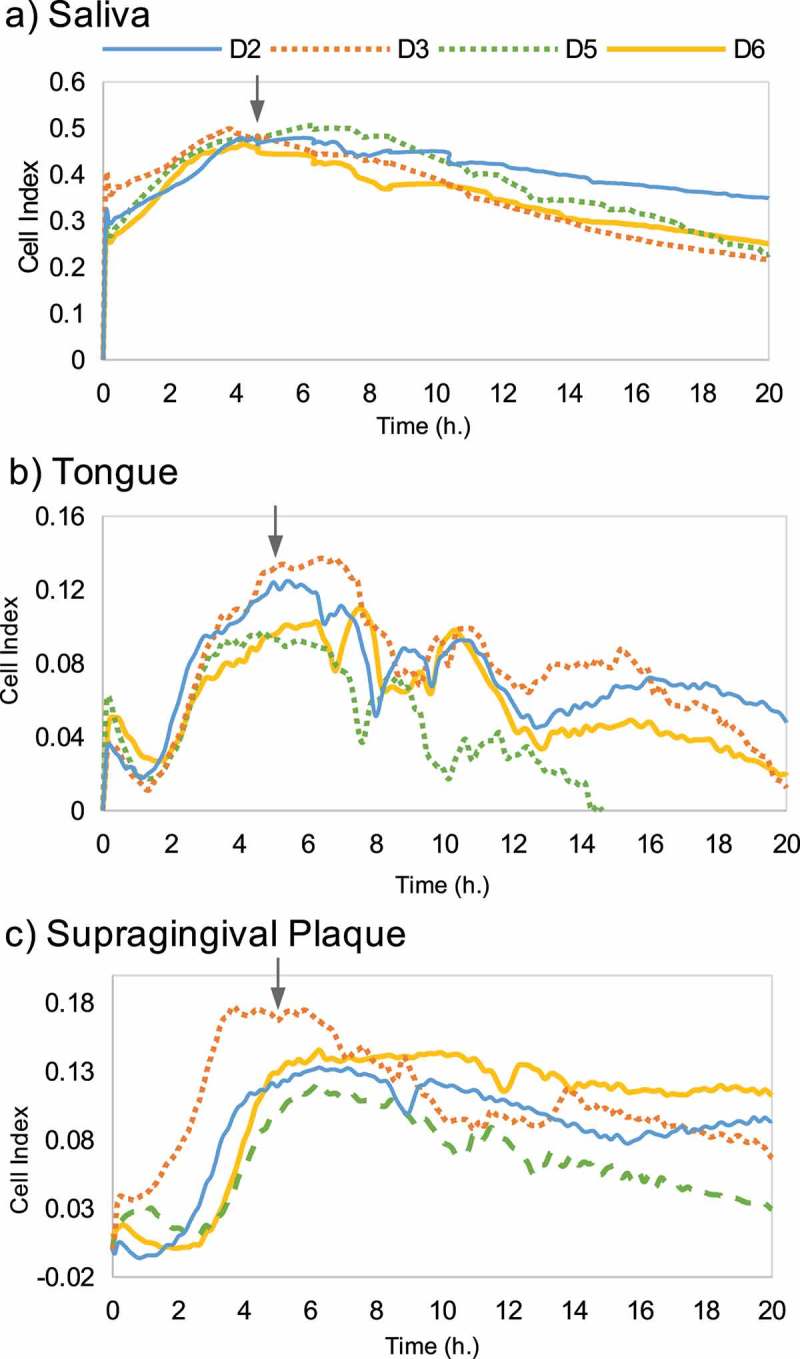
Biofilm formation dynamics of oral samples from three different niches (**a**) saliva, (**b**) tongue and (**c**) supragingival plaque, of four volunteers (D2, D3, D5 and D6). Arrows indicate the timepoint at which adhered biofilms were collected for DNA extraction and analysis of bacterial composition.

Complex biofilm formation of oral samples from three different niches (saliva, tongue and supragingival plaque) was monitored to find out whether the biofilm bacterial composition was conditioned by the inoculum type used. Curves in Figure 3 represent real-time CI values obtained when using each sample type in four individuals (D2, D3, D5 and D6). As shown in Figure 3, bacterial cells from all three types of samples (saliva, tongue and supra-gingival plaque) were able to grow attached to the wells and produced a measurable signal. However, the patterns of biofilm formation dynamics varied depending on the sample origin and were similar for the same sample type from different individuals.

**Figure 4.**
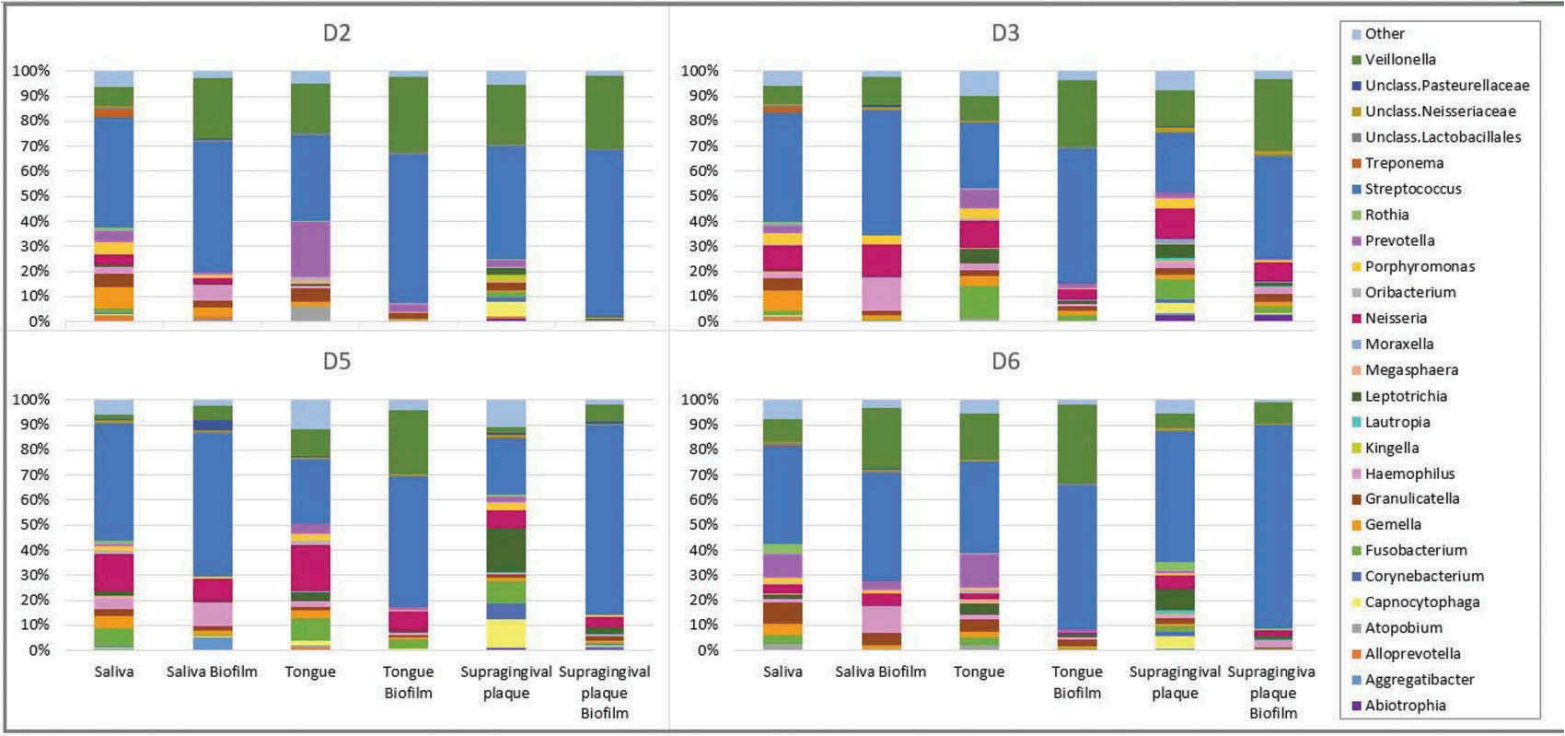
Bacterial relative abundance for each inoculum of the three different oral niches (saliva, tongue and supragingival plaque) as well as its respective biofilm in every of the four individuals (D2, D3, D5 and D6). The full dataset including the relative proportions of each bacterial genus for the different donors and biofilm types is provided in Supplementary Table 1.

**Figure 5.**
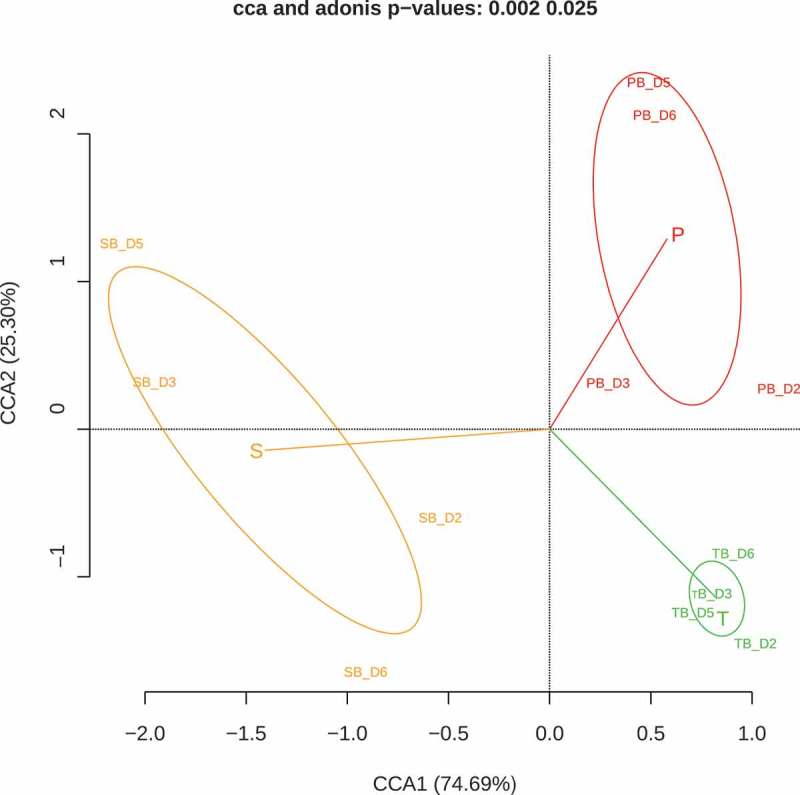
Canonical Correspondence Analysis (CCA) of microbial community composition of biofilm samples from saliva (SB), tongue (TB) and subgingival plaque (PB) of four volunteers (D2, D3, D5 and D6).

When the biofilms reached stationary phase, the bacterial composition of adhered cells was analyzed. Figure 4 shows the bacterial relative abundance of each inoculum and its respective biofilm of the four individuals. Broadly, under the growth conditions tested, the abundance of *Veillonella* and *Streptococcus* was increased in the biofilm samples when compared with their original inoculum, at the expense of a decrease in the genera *Prevotella, Neisseria, Fusobacterium* and *Aggregatibacter*. The relative abundances of all bacterial genera in each sample (inoculum and biofilm) are shown in Supplementary Table 1. Canonical Correspondence Analysis (CCA) was used to investigate the correlation between inoculum origin and the observed microbial community composition. Each circle (Figure 5) represents a different bacterial community structure from a specific sample type (saliva [S], tongue [T] and supragingival plaque [P]), and biofilm samples group together according to inoculum origin (Adonis value = 0.002, *p*= 0.025), reflecting their similarity degree in bacterial composition. In addition, biofilms and their corresponding original inoculum samples also grouped together (Figure S1) and in a different cluster for every niche type (Adonis value = 0.001, *P*= 0.045). These results indicate that bacterial composition of the complex biofilms formed in the RTCA wells is intimately linked to the sample from which it is collected, even though the composition is not identical to the inoculum used under the culture medium and growth conditions used.

**Figure 6.**
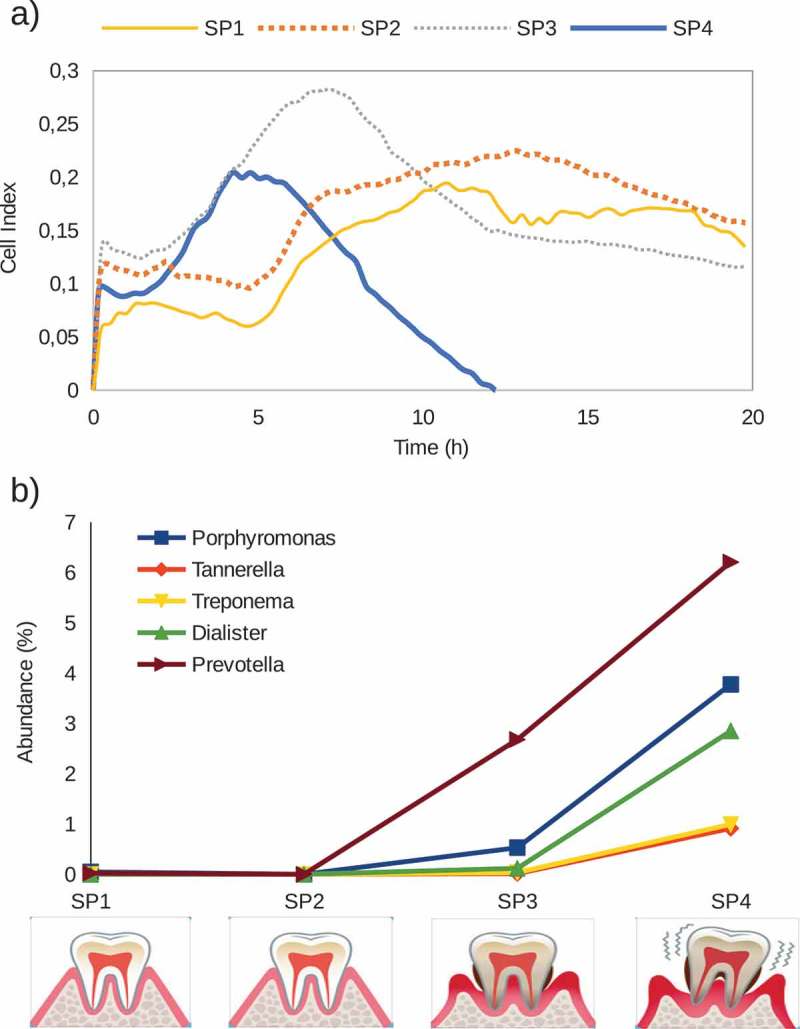
(**a**). Biofilm formation, as indicated by impedance Cell Index values, of subgingival plaque samples from four donors (SP1, SP2, SP3 and SP4). Clinical examination of the patients indicated that SP1 and SP2 had healthy gums, SP3 presented gingivitis and SP4 suffered from periodontitis (periodontal pockets depth >5 mm). Gingival health status of the donors is represented by illustrative images in the lower part of the figure. (**b**) Relative abundance of periodontopathogens in the formed biofilms of the four different subgingival plaque samples.

Another oral niche with important clinical implications is the subgingival plaque, due to its role in gingivitis and periodontal disease. A pilot test was conducted using subgingival samples from four patients with different periodontal health status. Figure 6(a) presents biofilm formation CI data of subgingival plaque samples of those patients (SP1, SP2, SP3 and SP4), whose clinical records showed a decreasing degree of periodontal health, ranging from healthy gums to gingivitis and periodontitis with deep periodontal pockets. Interestingly, the patterns of biofilm formation varied according to periodontal health status, showing faster and shorter biofilm dynamics in patients with periodontal disease. To investigate a potential correlation between periodontal health and the levels of periodontal disease-associated pathogens in the *in vitro* biofilms, bacterial composition was determined by *16S rDNA* Illumina sequencing, allowing us to report the relative abundance of periodontopathogens in the formed biofilm. Figure 6(b) shows periodontal pathogens’ abundance in the four *in vitro* biofilms derived from subgingival plaque samples. Each patient is represented on the X-axis (SP1, SP2 SP3 and SP4), under which an illustration depicts periodontal health status. In biofilms formed by subgingival plaque samples from patients with healthy periodontium (SP1 and SP2), periodontal pathogens are not detected; by contrast, in biofilms samples of patients SP3 and SP4, an abundance of these pathogens increased. To confirm these results, subgingival plaque samples from periodontal pockets of 10 patients with ‘chronic’ periodontitis were collected and used as inocula for biofilm growth. The results show the growth of all major periodontal pathogens in the *in vitro* biofilms obtained, including the three organisms from the ‘red complex’ of periodontal disease (Figure S2, published as supplementary material). Although preliminary, the data strongly indicate the growth of multiple-species, complex biofilms whose bacterial composition is intimately related to the microbial diversity of their inoculum, highlighting the potential of the system as a representative *in vitro* model of oral biofilms with different applications. One of those applications is the study of antibiotic sensitivity in complex biofilms.

### Amoxicillin effect on oral biofilms

**Figure 7.**
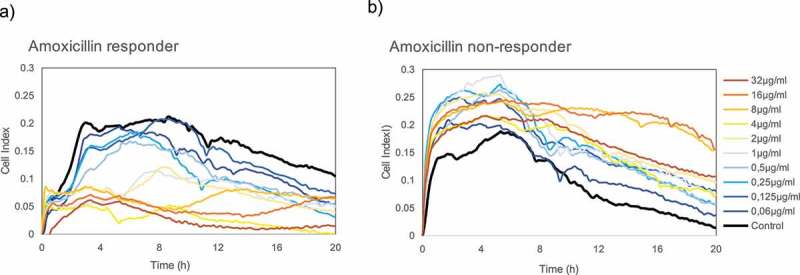
Real-time growth measurements of saliva-derived biofilms in the presence of different doses of amoxicillin in two different volunteers. (**a**) Donor in which the saliva-derived biofilm appeared to be sensitive to the antibiotic (amoxicillin ‘responder’); (**b**) Donor in which the saliva-derived biofilm appeared to be resistant to the antibiotic (amoxicillin ‘non-responder’), inducing biofilm formation.

Amoxicillin with clavulanic acid is the most frequently used antibiotic for the treatment of dental infections. However, its antibacterial activity is normally tested on single species, and data on its ability to prevent multiple-species biofilm formation are scarce. To illustrate the potential of the RTCA system to evaluate the antibiotic sensitivity of oral biofilms, a pilot test was performed on biofilms grown from saliva from two different volunteers. Figure 7 shows real-time dose–response experiments in the two donors, one of which appears to be an amoxicillin responder (Figure 7(a)) and the other one a non-responder (Figure 7(b)). By this, we mean that the saliva-derived *in vitro* biofilm appears to be sensitive or resistant, respectively, to this antibiotic. In the amoxicillin responder, antibiotic concentrations equal to or greater than 2 μg/ml were capable of inhibiting or decreasing biofilm formation over 50% relative to the control. In the amoxicillin non-responder, on the other hand, there was no concentration able to inhibit biofilm formation, and in fact, all antibiotic dilutions *increased* its growth, suggesting that this antibiotic could, in fact, stimulate biofilm growth. In order to confirm these results, saliva samples from five other individuals were tested for biofilm growth in the presence of 8 μg/ml amoxicillin (the maximum expected plasma concentration after antibiotic administration). Biofilm growth curves are shown in Figure S3 and indicate that, under the conditions tested, the antibiotic was able to inhibit biofilm growth in some of the cases (Figure S3e), whereas in other individuals it had no effect (Figure S3b) or it actually induced higher biofilm growth (Figure S3a).

## Discussion

It is well established that under free-living conditions almost all microorganisms do not live isolated in suspension but coexist interacting with each other to form complex communities adhered to living or inert surfaces [34]. Likewise, the behavior of an individual bacterium in planktonic life is greatly modified as soon as it becomes part of a community with a unique neighborhood of its own species (mono-species biofilm), and even more so when the community is heterogeneous in composition (complex or multi-species biofilm) with a wide range of engagements between its constituents [35]. Oral biofilms are also known to have multiple levels of synergistic and antagonistic interactions [36] and therefore studying individual oral species in pure culture does not take into account the complex ecological interactions that take place in biofilms [37]. In the current work, we present a new model to study oral biofilm formation in real time. This approach has several advantages over current ones, including minimal sample handling and biofilm manipulation; fast and real-time monitoring of results; a correlation in microbial composition between the sampled microniche and the obtained biofilm; and the possibility to study the dynamics of biofilm formation.

The RTCA system enables one to observe the different phases of biofilm formation in real-time. Single-species biofilms such as *S. mutans* biofilm, for instance, developed for a period of 24 h (Figure 1), whereas complex biofilms derived from saliva samples reached stationary phase in just a few hours (Figure 2), suggesting multiple synergistic interactions among members of the oral biofilm [36–38]. The effect of compounds which are vital for biofilm formation can also be evaluated. For example, sucrose addition to the medium, increased biofilm growth in *S. mutans* more than six-fold (Figure 1), likely reflecting the transformation of sugar into glucans with which this bacterium adheres to hard surfaces [39]. In the case of complex biofilms, post-meal saliva samples resulted in an extension of the stationary phase before the disassembly step (Figure 2). In this regard, identifying and discerning the different biofilm development steps could provide valuable information for studying biofilm growth, as well as for the design of new treatment strategies focused on blocking or inhibiting specific steps. It has also to be kept in mind that a decrease in CI could be due not only to biofilm disassembly but also to any alteration in impedance as a consequence of changes in compounds that could influence the conductivity of the culture medium. For instance, some antibiotics appear to alter impedance and therefore appropriate controls are always needed [23]. Thus, although a relationship between biofilm mass and CI values was clearly established for single-species biofilms [20] future experiments should confirm that decreases in CI values correspond to biofilm disaggregation and not to artefactual changes in conductivity.

Although performed with a limited number of samples, the biofilm growth curves from the same sample type (tongue, saliva, etc.) derived from different individuals follow similar formation dynamics. In the case of saliva biofilms, a fast growth is observed in the first h of development, under both aerobic (Figure 2(a,b)) and anaerobic conditions (Figure 3(a)), possibly reflecting the fast pellicle formation by salivary glycoproteins followed by bacterial attachment [2]. On the other hand, biofilms derived from tongue samples displayed a characteristic pattern with growth dynamics that resemble a sawtooth pattern, showing successive phases of biofilm growth and disintegration (Figure 3(b)). To our knowledge, this oscillating pattern of biofilm growth has not previously been reported in oral biofilms, but this preliminary finding should be confirmed by larger sample sizes, and future studies should determine if it is due to inter-cellular signaling like Quorum Sensing mechanisms, which are common in oral bacteria [40], or whether it is an artifact of the *in vitro* conditions which do not reflect biofilm growth *in vivo*. Supragingival dental plaque biofilms (Figure 3(c)) displayed fewer and less dramatic oscillations. Biofilms from subgingival samples did not show this oscillating pattern and appeared to show different patterns depending on periodontal health status (Figure 6). The sequencing data presented in the current manuscript show a good degree of correlation between bacterial composition in the biofilms and their corresponding sample (Figure 5). The growth conditions are nevertheless crucial, including the aerobic/anaerobic atmosphere or the culture medium. Under the conditions tested in the current manuscript, some bacteria appear to be favored, such as streptococci (Figure 4), a feature which is also frequently seen in other *in vitro* models of oral biofilms [41]. However, the system allows the growth of strict anaerobes and fastidious organisms like those present in periodontal pockets (Figure 6), including the TM7 phylum [42]. Thus, the association of biofilm formation dynamics with sample type, together with the DNA sequencing data showing that the composition of the *in vitro* biofilms is also sample-dependent, open a wide range of possibilities for the study of multiple-species oral biofilms. These include the use of the system to better understand biofilm growth and dynamics, to search for and test new molecules that could modulate biofilm growth, or as a tool for deciding individual-based therapeutic treatments. Moreover, the xCELLigence device could also be used to determine the *in vitro* effectiveness of commonly prescribed antibiotics for odontogenic diseases treatment and to select, in less than 24 h, the specific one for each patient that increases the chance of success *in vivo*, as well as a great tool for screening new substances with anti-biofilm effects.

The low reported effectiveness of antibiotic therapy on biofilms has been proposed to be a consequence of several reasons such as the low penetration of antimicrobial compounds through the biofilm matrix or the reduction of bacterial metabolism in ‘persister’ cells, among others. In agreement with this, the required antibiotic dose found to be needed to disintegrate a mature *S. mutans* biofilm was 240-fold higher than that necessary to inhibit its formation (Figure 1(b)). It must be kept in mind that antibiotic sensitivity tests are routinely carried out on pure cultures grown in a planktonic state like microdilution methods or through agar diffusion tests, where the measured minimum inhibitory concentrations are very different from those obtained when the microbes are grown on biofilms [20]. Taking into account that more than 80% of microbial infections in the human body are due to bacteria forming biofilms [43], an improvement of the methods to evaluate antibiotic susceptibility is instrumental to achieve higher treatment effectiveness, to individualize the treatments and to decrease bacterial resistance. The situation is also critical for infections produced by multiple-species biofilms such as those involved in oral diseases, where the effectiveness of a given antibiotic treatment is even more difficult to foresee due to potential synergism and interrelationships of its components and due to the lack of valid *in vitro* models where complex biofilms can be tested. The RTCA system presented in the current manuscript could contribute to improve antibiotic susceptibility tests in those cases. Although preliminary, the cases shown in Figure 7 illustrate the different effects of amoxicillin on oral biofilm formation from two volunteers. In this case, the same antibiotic concentration (32 μg/ml) resulted in opposite effects, namely biofilm inhibition (Figure 7(a), amoxicillin responder) or induction (Figure 7(b), amoxicillin non-responder), a pattern which was confirmed in other donors (Figure S3). These results support the view that, to be truly effective, antibiotic selection should be led towards a personalized therapy where antibiotic susceptibility tests are performed on an individual bases with complex samples. Recently, it has been proposed that diagnostic, preventive and therapeutic strategies in dentistry should be personalized [44,45]. We hope the current manuscript will initiate further studies that could on one hand search for new biofilm-disrupting or inhibitory compounds, and on the other hand implement real-time monitoring systems to individualize antibiotic treatment in dentistry.
