# Supplementary material for: Development of an in vitro system to study oral biofilms in real time through impedance technology: validation and potential applications
Source: J Oral Microbiol. 2019 May 6;11(1):1609838. doi: 10.1080/20002297.2019.1609838 (PMC6507917; doi:10.1080/20002297.2019.1609838)
Supplement: Supplemental Material [file ZJOM_A_1609838_SM1672.zip › supplemental data/Supplemental data.docx]

**Supplementary Figures**

**Figure S1.** Canonical Correspondence Analysis (CCA) of microbial community composition of biofilm samples from saliva (SB), tongue (TB) and subgingival plaque (PB) of the four volunteers (D2, D3, D5 and D6) and the inoculum from which they originated, respectively (S, T and B).

**Figure S2 a**). Biofilm formation (measured as Cell Index values) of subgingival plaque samples from pockets of patients with periodontitis (n = 10) grown for 12 h. The black arrow at 7 h indicates the timepoint in which biofilm samples were collected. **b**) Bacterial composition, as measured by Illumina sequencing of the *16S rRNA* gene, of subgingival plaque samples used as an inoculum and their corresponding biofilms after 7 h of growth. ‘Red-complex’ genera (containing bacteria strongly associated with periodontitis), are marked with red arrows.

**Figure S3.** Real-time growth measurements (as indicated by Cell Index values) of saliva-derived biofilms in the presence of amoxicillin at 8 μg / ml (the plasma concentration after antibiotic administration) in five different volunteers.

**Supplementary Table 1.** Bacterial composition, as measured by Illumina sequencing of the *16S rRNA* gene, in oral samples and their corresponding *in vitro* biofilms from four donors (D2, D3, D5 and D6). Data show the relative abundances of all bacterial genera in each sample (inoculum and biofilm), including unstimulated saliva, tongue scrapings and supragingival dental plaque.
