# Supplementary figures and images for: Development of an in vitro system to study oral biofilms in real time through impedance technology: validation and potential applications
Source: J Oral Microbiol. 2019 May 6;11(1):1609838. doi: 10.1080/20002297.2019.1609838 (PMC6507917; doi:10.1080/20002297.2019.1609838)

Figure S1

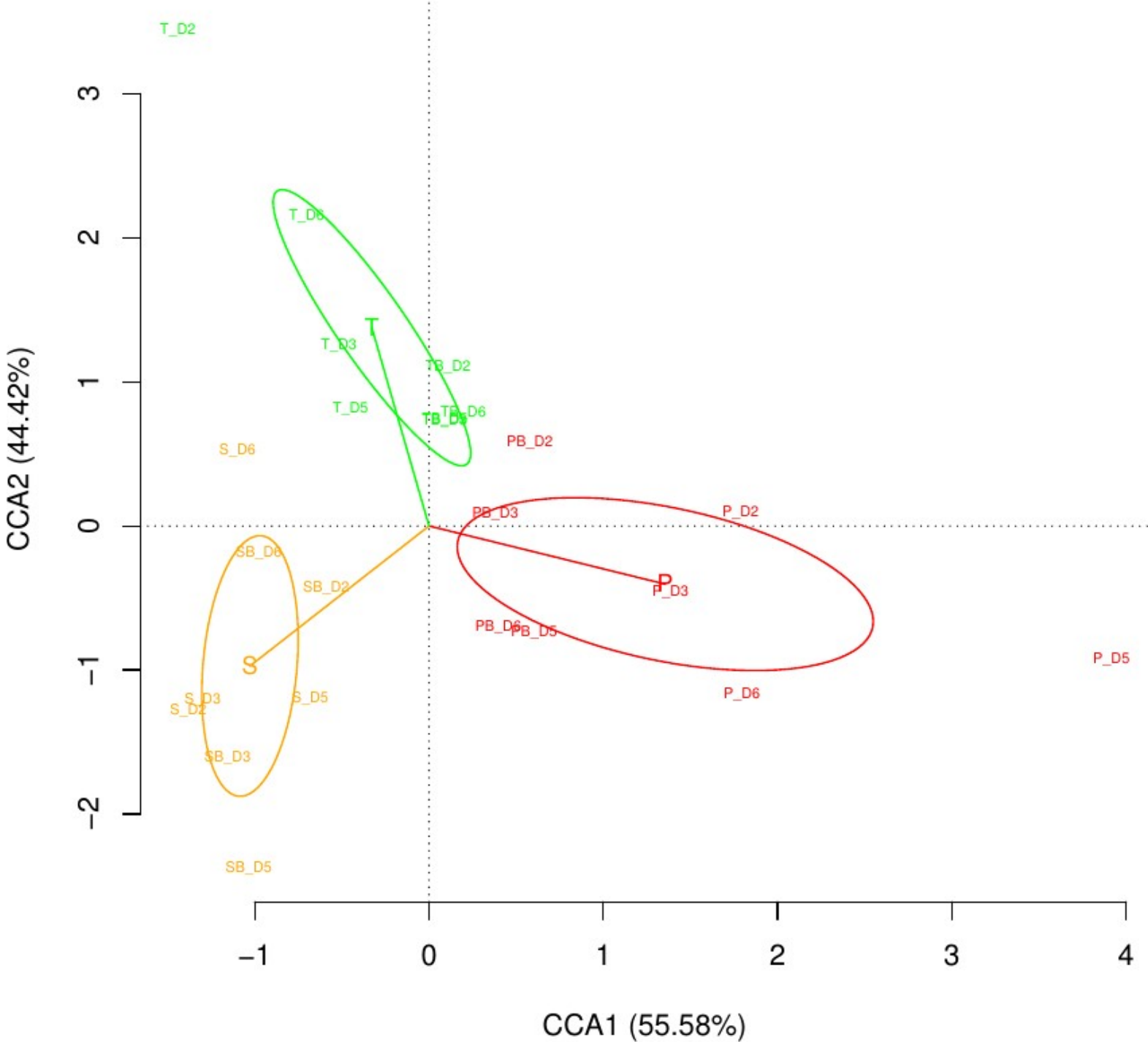

Supplement: Supplemental Material [file ZJOM_A_1609838_SM1672.zip › supplemental data/Figure_S1.pdf]

a)

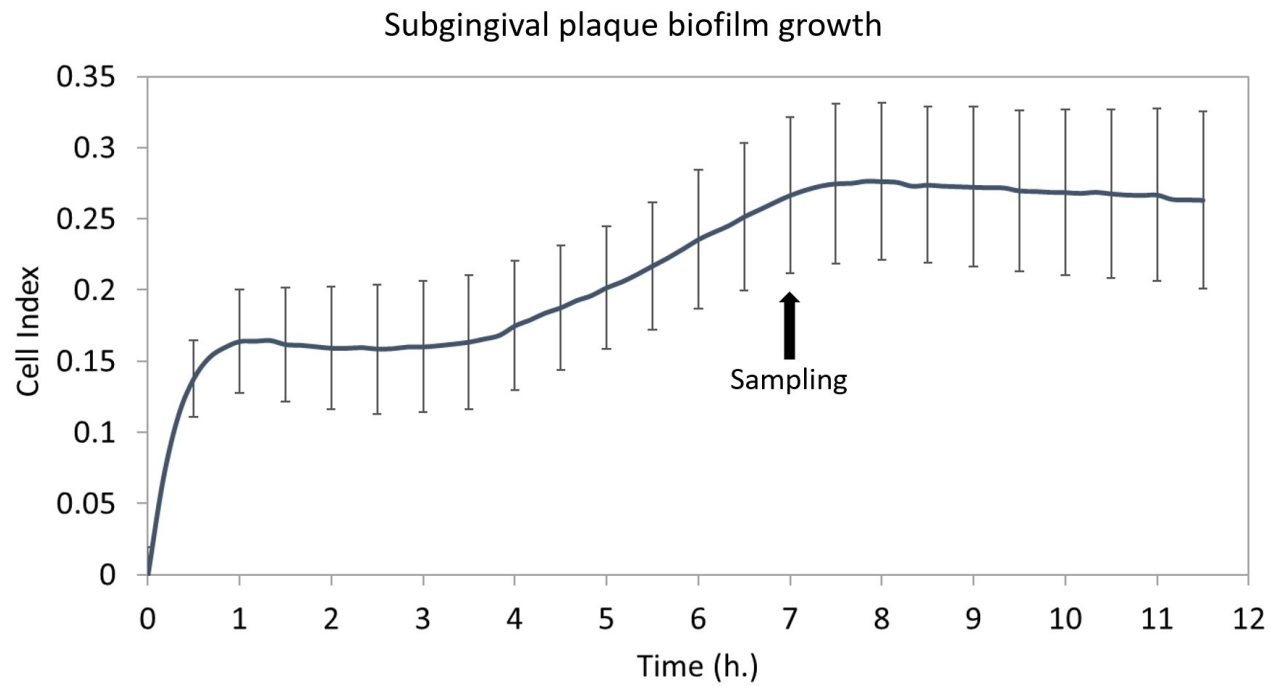

b)

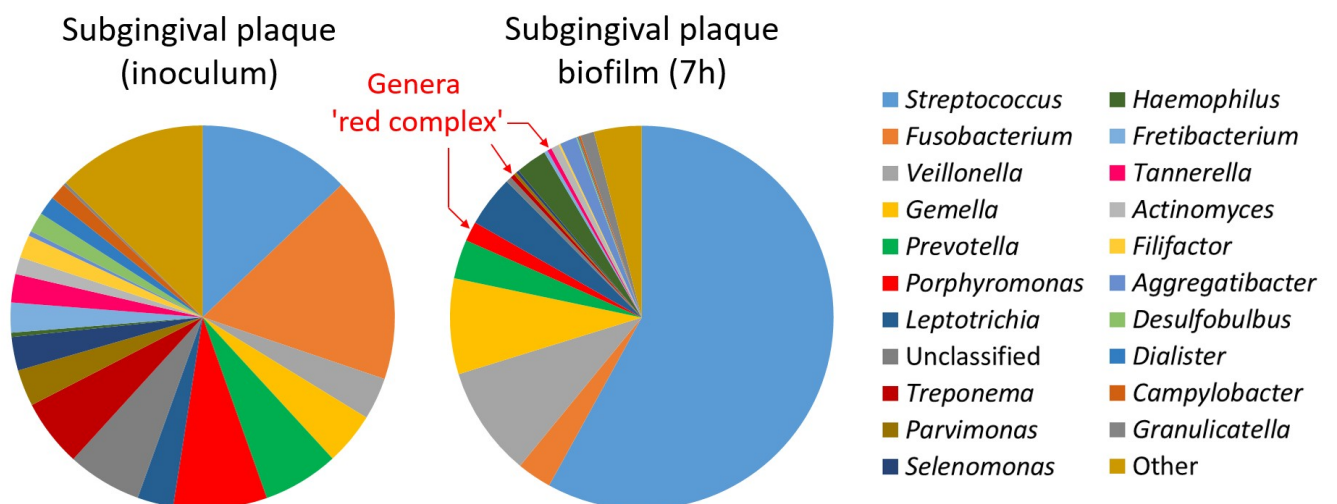

Supplement: Supplemental Material [file ZJOM_A_1609838_SM1672.zip › supplemental data/FIGURE_S2.pdf]

Figure S3

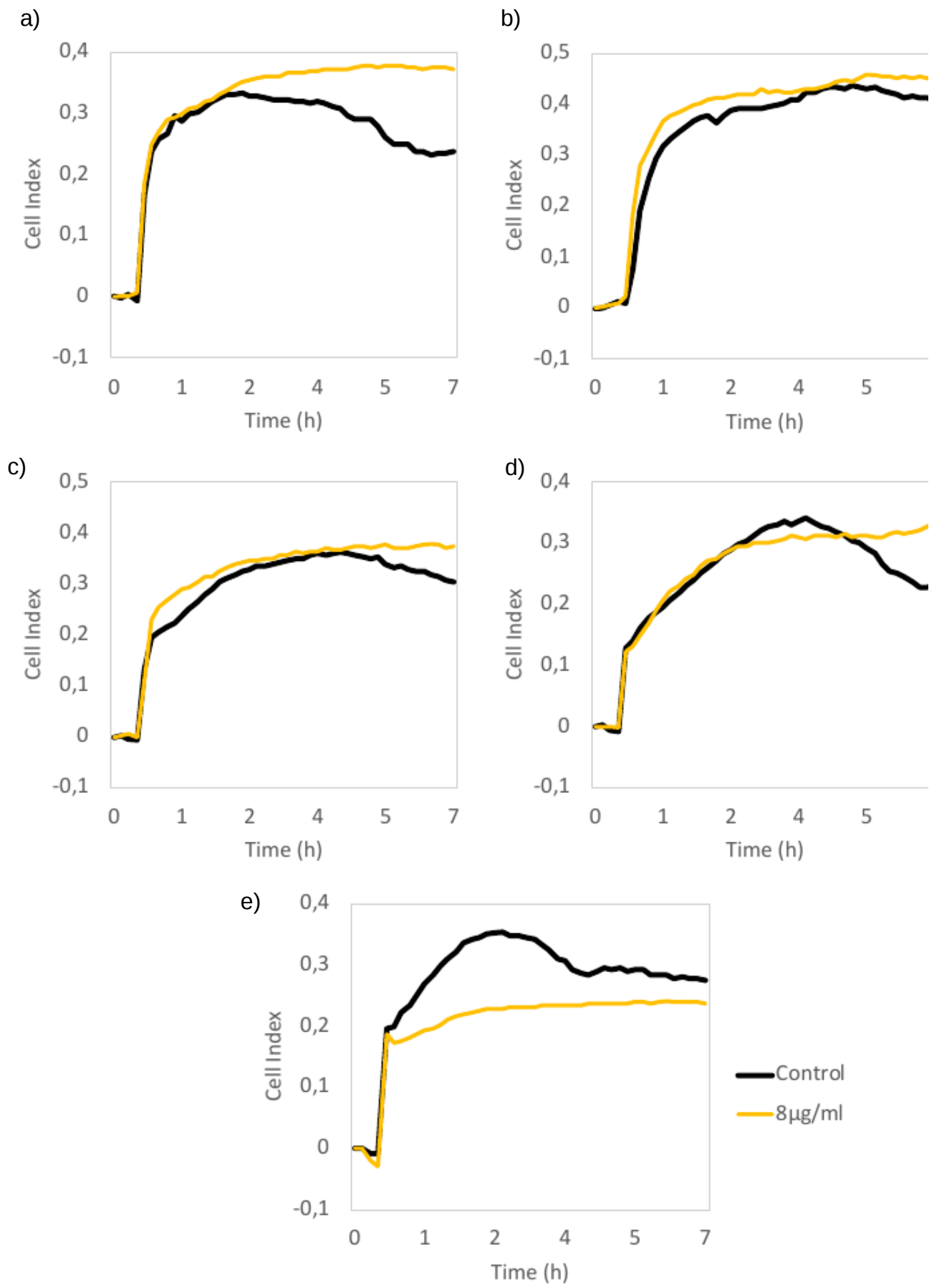

Supplement: Supplemental Material [file ZJOM_A_1609838_SM1672.zip › supplemental data/Figure_S3_final.pdf]
